# Profile of Older Adults Who Received Wound Care by a Family Caregiver: The NHATS-NSOC 2017

**DOI:** 10.1093/geroni/igaa057.2877

**Published:** 2020-12-16

**Authors:** Zachary Hathaway, Janeway Granche, Martha Coates, Justine Sefcik, Michael Neidrauer, Peter Lewin, Rose Ann DiMaria-Ghalili

**Affiliations:** Drexel University, Philadelphia, Pennsylvania, United States

## Abstract

A gap in knowledge exists related to the socio-demographic and health characteristics of older adults receiving wound care from a family caregiver in the home. We created a cohort (N=992) of older adults from NHATS who lived in the community or residential care (non-nursing home) and had a family caregiver complete the NSOC question “provides help with skin care related to wounds or sores”. Approximately one third (32%) of these older adults received wound care from a family caregiver. These older adults were more likely to be men, live with others, have lower levels of physical function, be malnourished (OR = 1.63 [95% CI = 1.02-2.60]), and have inflammation (hsCRP > median 1.89), P < .05. These findings can inform the needs of older adults receiving wound care from a family caregiver and lead to development of additional supports for caregivers (e.g., multi-component interventions).

